# Serum Levels of Zinc, Copper and Ferritin in Patients with Salivary Gland Tumors

**DOI:** 10.31557/APJCP.2019.20.2.545

**Published:** 2019

**Authors:** Zohreh Jaafari- Ashkavandi, Bijan Khademi, Mahyar Malekzadeh, Zeinab Shahmoradi

**Affiliations:** 1 *Oral and Dental Disease Research Center, Department of Oral and Maxillofacial Pathology, School of Dentistry,*; 2 *Otolaryngology Research Center, Department of Otolaryngology, *; 3 *Institute of Cancer Research, School of Medicine, Shiraz University of Medical Sciences, Shiraz, Iran.*

**Keywords:** Zinc, copper, ferritin, trace element, serum, salivary gland, tumor, cancer, pleomorphic adenoma

## Abstract

**Background:**

Variation in serum levels of trace elements including zinc, copper and ferritin has been reported in cancer patients. The aim of this study was to evaluate these trace elements in the patients’ sera with benign and malignant salivary gland tumors (SGTs) and compare them with normal individuals.

**Methods:**

In this cross-sectional study, 60 patients with SGTs including 16 pleomorphic adenoma and 44 malignant SGTs, as well as 28 healthy controls, were enrolled. Serum levels of zinc, copper and ferritin were determined by atomic absorption and ELISA methods. Data were analyzed using one way ANOVA, Chi-square, Kruskal-Wallis and Mann- Whitney tests.

**Results:**

The mean concentration of zinc, copper, ferritin was1.5± 2 ppm, 1.2± 0.5 ppm, and 96.7± 65.7 ng/ml in PA, 1.5± 1.4,1.3± 0.4, and 111.2± 112 in malignant SGTs, and1.1±0.3, 1.2± 0.23 and 124±135.8 in normal control groups. There was no statistically significant difference between the patients and control groups, and between benign and malignant SGTs (P>0.05).

**Conclusion:**

The serum levels of trace elements in SGTs were not different from normal individuals. The results might have been affected by some interventional factors. Therefore, designing cohort complementary studies might result in obtaining more accurate data.

## Introduction

Salivary gland tumors (SGTs) account for 3-6% of head and neck cancers. The most common benign SGT is pleomorphic adenoma (PA) and the malignant ones are adenoid cystic carcinoma (AdCC) and mucoepidermoid carcinoma (MEC). Irradiation, tobacco and nutritional factors have been reported as etiologic factors, but the exact mechanism is still unknown (Guzzo et al., 2010; Su et al., 2015). Some trace elements, such as iron, zinc (Zn) and copper (Cu), play a critical role in some pathological processes due to their presence in the structure of many enzymes, such as antioxidant enzymes (Zablocka-Slowinska et al., 2018). The reduced function of antioxidant enzymes due to imbalance of trace elements often results in oxidative damages (Huang et al., 2018). Oxidative stress might contribute to cancer development due to production of toxic and mutagenic metabolites (Martinez-Useros et al., 2017).

Zn is an important element which plays a role in a large number of metaloenzymes (Andreini and Bertini, 2012). In addition, it is considered as antioxidant component of metaloproteinases (Sharif et al., 2012). It is reported that serum Zn levels decreased or increased in some human cancers, such as lung, colorectal, renal, bladder and oral ones (Mao and Huang, 2013; Pirincci et al., 2013; Baharvand et al., 2014a; Khoshdel et al., 2016; Zablocka-Slowinska et al., 2018).

Cu is an essential element and its concentration is elevated in cancer tissues, which promotes cancer development via some mechanisms, such as increasing angiogenesis (Wang et al., 2010).Tumor progression and metastasis initiated along with the increasing Cu serum levels in patients with lymphoma and breast cancer (Jamakovic and Baljic, 2013; Pavithra et al., 2015). Also, Cu/Zn ratio is stated to be effective in diagnosis of some cancers. In some studies, Zn/Cu ratio has been used to evaluate the prognosis of cancer patients (Golabek et al., 2012; Malavolta et al., 2015).

Ferritin, iron storage protein, stored in the human liver, spleen and bone marrow, reflects the amount of iron in the body. Ferritin makes iron available for critical cellular processes, while protecting lipids, DNA, and proteins from the potentially toxic effects of iron serum (Alkhateeb and Connor, 2013; Baharvand et al., 2014b). Ferritin levels change in the inflammatory diseases as well as in some malignant tumors (Alkhateeb and Connor, 2013).

Since the role of micronutrients in SGTs is relatively unknown, this study aimed to evaluate Zn, Cu and Ferritin serum levels in patients with benign and malignant salivary gland tumors.

## Materials and Methods

This cross-sectional study was approved by the Ethics Committee of Shiraz University of Medical Sciences (IR.sums.rec.1395.S1016). Sixty patients were enrolled in this study, including16 patients with PA, 44 patients with malignant SGTs (18 cases of MEC, 10 ADCC, 4 acinic cell carcinoma (ACC), 4 squamous cell carcinoma (SCC), 8 malignant mixed tumor), as well as 28 normal subjects. The diagnosis was confirmed for all patients histopathologically. The patients with any anti-neoplastic treatment or a history of other malignancies were excluded. The patients and normal controls did not have any history of other systemic diseases and complementary drug consumption. All subjects gave their written informed consent. Baseline data including the patients’ age, gender and type of tumor were obtained from their medical files.

Before any therapeutic intervention, 5 ml blood samples were collected in tubes containing EDTA and centrifuged. The serum was separated and transferred to acid wash tubes and kept at -80ºC for further analysis.

The metal concentrations (Zn and Cu) were assessed by atomic absorption spectrophotometry (spectrophotometer type AAS1, Karl Zeiss, Jena, Germany).

Ferritin level was measured using enzyme linked immunosorbent assay (ELISA) kit (Monobind, USA). The mean Zn, Cu and ferritin serum levels, as well as Cu/Zn, in patients and control groups were analyzed and compared through SPSS (version 11), using Chi-square, one-way ANOVA, Kruskal-Wallis and Mann–Whitney tests.

## Results

Baseline data of all groups are shown in Table 1. As shown, the mean serum Zn, Cu and ferritin levels, as well as Cu/ Zn, in all groups are shown in Tables2, 3.Moreover, normal or abnormal levels of trace elements in all study groups are illustrated in Table 4.

The mean serum Zn levels were 1.5± 2, 1.5± 1.4, and 1.1±0.3 ppm in the PA and malignant tumors and control group, respectively. Cu and ferritin levels were 1.2± 0.5 ppm and 96.7± 65.7 ng/ml in the PA patients; 1.3± 0.4 and 111.2± 112 in malignant tumors; and 1.2± 0.23 and 124±135.8 in normal individuals. Statistical analysis using one-way ANOVA test showed that the mean serum Zn, Cu and ferritin levels were not statistically different among the study groups (P> 0. 05).

**Table 1:** Baseline Data of All Study Groups

Tumor type	Age	M/F	Stage
	Mean±SD		?/I/II/III/IV
Benign: PA (16)	43.8±13.7	6/10	_
Malignant: MEC (18)	54.2±18.4	8/10	4/2/3/3/6
ADCC (10)	48.2±19.4	5/5	7/0/1/1/1
Other (16)	50.9±16	13/3	0/11/0/1/3/1
Control (28)	47.2±14.3	15/13	_
Total	47.8±16.4	47/41	11/13/5/7/8

?, Undefined stage; PA, pleomorphic adenoma; MEC, mucoepidermoid carcinoma; ADCC, adenoid cystic carcinoma; Other, 4 acinic cell carcinoma; 4 squamous cell carcinoma, 8 malignant mixed

**Table 2:** Mean of Ferritin, Cu and Zn Groups Serum Levels in Patients and Control Groups

Tumor Type	Ferritin (ng/ml)	Cu (ppm)	Zn (ppm)
Benign: PA (16)	96.7±65.7	1.16±0.48	1.50±2
Malignant: MEC (18)	66.1±72.1	1.39±0.3	2.90±4.1
ADCC (10)	137.7±137	1.44±0.6	1.22±0.3
ACC (4)	96.1±32.7	1.22±0.5	1.1±0.4
SCC (4)	121.4±100.5	0.97±0.4	1±0.2
Malignant mixed tumor (8)	163.5±159.1	1.15 ±0.4	2.5±2.2
Control (28)-	123.9±135.9	1.17±0.2	1.09±0.36

PA, pleomorphic adenoma; MEC, mucoepidermoid carcinoma; ADCC, adenoid cystic carcinoma; ACC, acinic cell carcinoma; SCC, Squamous cell carcinoma

**Table 3:** Cu/ Zn Ratio in All Study Groups

Tumor Type	Cu/Zn
Benign: PA (16)	1.03±0.3
Malignant: MEC (18)	1.5±1. 8
ADCC (10)	1 ±0.4
ACC (4)	1.1±0.4
SCC (4)	1.03±0.6
Malignant mixed tumor (8)	0.8 ±0.4
Control (28)-	1.1±0.3

PA, pleomorphic adenoma; MEC, mucoepidermoid carcinoma; ADCC, adenoid cystic carcinoma; ACC, acinic cell carcinoma; SCC, Squamous cell carcinoma

**Table 4:** Zn, Cu and Ferritin Status in All Study Groups

Group (N)	Zn	Cu	Ferritin
	Low/Normal/High	Low/Normal/High	Low/Normal/High
PA (16)	0/12/3	11/2/3	0/15/1
Malignant (44)	0/27/17	2/30/12	4/36/4
Control (28)	0/23/5	0/24/4	4/18/6
Total (88)	0/62/25	13/56/19	8/69/11

Normal ranges: Zn, 0.7-1.1, Cu: 0.75-1.45; Ferritin: Male, 12-300; Female, 12-150; PA: pleomorphic adenoma

As shown in Table 4, most of the patients and normal individuals exhibited normal Zn level. Although the percentage of normal Zn level in the control group (82%) and benign tumors (75%) was more than that in malignant ones (61.4%), Chi-square test showed no significant difference among the groups. Cu and ferritin levels presented a similar pattern and most of the subjects revealed a normal level of these elements. Chi- square test showed no difference among the groups regarding to the status of Cu and ferritin levels. (All p- values >0.05)

Cu/Zn ratio was 1± 0.3, 1.1± 0.5 and 1.1± 0.3 in PA, malignant tumors and control groups, respectively. Kruskal-Wallis test show no difference among the study groups (p> 0.05).

## Discussion

Researchers have revealed some relationships between the levels of trace elements and the tumor type, stage or activity. In the present study, we evaluated the serum levels of Zn, Cu and ferritin in patients with benign and malignant SGTs. The findings showed that the majority of patients expressed normal concentrations of these elements, which was not different from the normal individuals.

Zn is recognized as an antioxidant agent and plays an anticancer role by activating some transcription factors that control the cell proliferation and apoptosis as well as DNA, RNA and ribosome stability. It is also effective in DNA repair. Low Zn concentration has been reported in many cancers, such as lung, bladder, colorectal and renal carcinomas (Mao and Huang, 2013; Pirincci et al., 2013; Khoshdel et al., 2016; Zablocka-Slowinska et al., 2018). Another study (Gradinaru et al., 2007) reported low Zn concentration in a preliminary study in 31 patients with stage II and III malignant SGTs of the parotid glands. In this study, the plasma Zn level in patients was similar to our results; however, the control group showed a higher level of Zn. While Zn deficiency was reported in Iranian population (Ghasemi et al., 2012), it seems that serum Zn levels are related to dietary habits. It is shown that Zn supplementation might reduce DNA damage and inflammation sources (Sun et al., 2016). In addition, some studies have shown increased Zn levels in oral and testicular cancer patients (Baharvand et al., 2014a; Kaba et al., 2015).

Some researchers have proposed that evaluating Zn concentration in tissues is a more precise marker for cancer development. Hashemian et al., (2014) in a review study explained that the benefits of Zn supplementation in cancer incidence was not observed.

Although many researchers have observed increased Cu concentration in the sera of some cancer patients, such as testicular, oral, bladder (Mao and Huang, 2013; Baharvand et al., 2014a; Kaba et al., 2015), the present findings regarding SGTs did not reveal any difference in comparison to the normal subjects. One study reported lower Cu serum level in patients with colorectal cancer in comparison with normal individuals and found Cu deficiency in the population (Khoshdel et al., 2016). In Gardinaru et al.,’s study (2007), Cu exhibited a higher concentration in patients with malignant SGTs in comparison with the control group. Cu is an essential trace element, which is essential in several steps of cancer progression, and it elevates in cancer cells (Blockhuys et al., 2017). It was shown that the interaction between vitamin D and Cu leads to severe oxidative stress, DNA fragmentation and cell death, especially in malignant cells (Rizvi et al., 2015). Also, Cu is required for angiogenesis. Several studies have shown the tumor inhibitor effects of Cu chelating compounds (Santini et al., 2014). The lower and higher concentrations of Zn and Cu have been reported in different human cancers. The conflicting data regarding serum levels of trace elements could be attributed to the study design, sample size, dietary habits and racial factors. Most studies have reported the mean concentration of these elements in a total sample group, while the normal range of these elements are too wide and the patients might be diagnosed in the normal range even in a different mean levels.

The present study did not find any change in the ferritin serum levels in the patients compared to the control group. Although the role of intracellular ferritin has been well recognized, the significance of its serum level is poorly understood in cancer prevention(Alkhateeb and Connor, 2013). A higher serum level has been detected in some human cancers including breast and oral carcinomas (Alkhateeb et al., 2013; Baharvand et al., 2014a). It has been found that tumor-associated macrophages have expressed high levels of ferritin. Moreover, some cancer cells can secret ferritin. These elevated levels in the tumor environment and serum have correlated with more aggressive diseases and poor outcome. The elevation of ferritin level in cancer patients has been attributed to chronic inflammation (Alkhateeb and Connor, 2013; Alkhateeb et al., 2013). However, it has been shown that 90% of patients with non-small lung cancer, as well as patients with bladder cancer, showed lower serum ferritin levels (Kukulj et al., 2010; Mazdak et al., 2010). Ferritin might decrease as a result of anemia or tumor resection in cancer patients. Ferritin levels might be increased in anemic patients due to chronic inflammation (Rambod et al., 2008). Some studies reported the mean ferritin serum levels. However, this element has a wide and different normal range regarding the age and gender. Moreover, anemia might be prevalent in a population, which could affect the result.

In conclusion, based on the present findings, Zn, Cu and ferritin serum levels in patients with SGT were similar to normal subjects, in contrast to many human neoplasms. The results may be affected by the patients’ dietary habits, socioeconomic factors, sample size, measurements and study design. Therefore, designing cohort complementary studies might result in obtaining more accurate data. Moreover, various types of malignant SGTs were enrolled in this study which had different values of the evaluated elements. This variation could affect the results; therefore, further studies focusing on a specific type of malignant SGT with a greater sample size are suggested.

## Statement conflict of interest

There is no conflict of interest.

## References

[B1] Alkhateeb AA, Connor JR (2013). The significance of ferritin in cancer: Anti-oxidation, inflammation and tumorigenesis. Biochim Biophys Acta.

[B2] Alkhateeb AA, Han B, Connor JR (2013). Ferritin stimulates breast cancer cells through an iron-independent mechanism and is localized within tumor-associated macrophages. Breast Cancer Res Treat.

[B3] Andreini C, Bertini I (2012). A bioinformatics view of zinc enzymes. J Inorg Biochem.

[B4] Baharvand M, Manifar S, Akkafan R (2014a). Serum levels of ferritin, copper, and zinc in patients with oral cancer. Biomed J.

[B5] Baharvand M, Manifar S, Akkafan R (2014b). Serum levels of ferritin, copper, and zinc in patients with oral cancer. Biomed J.

[B6] Blockhuys S, Celauro E, Hildesjo C (2017). Defining the human copper proteome and analysis of its expression variation in cancers. Metallomics.

[B7] Ghasemi A, Zahediasl S, Hosseini-Esfahani F (2012). Reference values for serum zinc concentration and prevalence of zinc deficiency in adult Iranian subjects. Biol Trace Elem Res.

[B8] Golabek T, Darewicz B, Borawska M (2012). Copper, zinc, and Cu/Zn ratio in transitional cell carcinoma of the bladder. Urol Int.

[B9] Gradinaru I, Ghiciuc CM, Popescu E (2007). Blood plasma and saliva levels of magnesium and other bivalent cations in patients with parotid gland tumors. Magnes Res.

[B10] Guzzo M, Locati LD, Prott FJ (2010). Major and minor salivary gland tumors. Crit Rev Oncol Hematol.

[B11] Hashemian M, Hekmatdoost A, Poustchi H (2014). Systematic review of zinc biomarkers and esophageal cancer risk. Middle East J Dig Dis.

[B12] Huang Y, Zhang Y, Lin Z (2018). Altered serum copper homeostasis suggests higher oxidative stress and lower antioxidant capability in patients with chronic hepatitis B. Medicine (Baltimore).

[B13] Jamakovic M, Baljic R (2013). Significance of copper level in serum and routine laboratory parameters in estimation of outspreading of Hodgkin’s lymphoma. Med Arch.

[B14] Kaba M, Pirincci N, Yuksel MB (2015). Serum levels of trace elements in patients with testicular cancers. Int Braz J Urol.

[B15] Khoshdel Z, Naghibalhossaini F, Abdollahi K (2016). Serum copper and zinc levels among Iranian colorectal cancer patients. Biol Trace Elem Res.

[B16] Kukulj S, Jaganjac M, Boranic M (2010). Altered iron metabolism, inflammation, transferrin receptors, and ferritin expression in non-small-cell lung cancer. Med Oncol.

[B17] Malavolta M, Piacenza F, Basso A (2015). Serum copper to zinc ratio: Relationship with aging and health status. Mech Ageing Dev.

[B18] Mao S, Huang S (2013). Zinc and copper levels in bladder cancer: a systematic review and meta-analysis. Biol Trace Elem Res.

[B19] Martinez-Useros J, Li W, Cabeza-Morales M (2017). Oxidative stress: A new target for pancreatic cancer prognosis and treatment. J Clin Med.

[B20] Mazdak H, Yazdekhasti F, Movahedian A (2010). The comparative study of serum iron, copper, and zinc levels between bladder cancer patients and a control group. Int Urol Nephrol.

[B21] Pavithra V, Sathisha TG, Kasturi K (2015). Serum levels of metal ions in female patients with breast cancer. J Clin Diagn Res.

[B22] Pirincci N, Gecit I, Gunes M (2013). Levels of serum trace elements in renal cell carcinoma cases. Asian Pac J Cancer Prev.

[B23] Rambod M, Kovesdy CP, Kalantar-Zadeh K (2008). Combined high serum ferritin and low iron saturation in hemodialysis patients: the role of inflammation. Clin J Am Soc Nephrol.

[B24] Rizvi A, Chibber S, Naseem I (2015). Cu(II)-vitamin D interaction leads to free radical-mediated cellular DNA damage: a novel putative mechanism for its selective cytotoxic action against malignant cells. Tumour Biol.

[B25] Santini C, Pellei M, Gandin V (2014). Advances in copper complexes as anticancer agents. Chem Rev.

[B26] Sharif R, Thomas P, Zalewski P (2012). The role of zinc in genomic stability. Mutat Res.

[B27] Su Y-X, Roberts DB, Hanna EY (2015). Risk factors and prognosis for myoepithelial carcinoma of the major salivary glands. Ann Surg Oncol.

[B28] Sun J, Shen R, Schrock MS (2016). Reduction in squamous cell carcinomas in mouse skin by dietary zinc supplementation. Cancer Med.

[B29] Wang F, Jiao P, Qi M (2010). Turning tumor-promoting copper into an anti-cancer weapon via high-throughput chemistry. Curr Med Chem.

[B30] Zablocka-Slowinska K, Placzkowska S, Prescha A (2018). Serum and whole blood Zn, Cu and Mn profiles and their relation to redox status in lung cancer patients. J Trace Elem Med Biol.

